# Model Systems of Gammaherpesvirus Infection, Immunity, and Disease

**DOI:** 10.1002/jmv.70581

**Published:** 2025-09-09

**Authors:** Arundhati Gupta, Renfeng Li, Kathy Shair, Shou‐Jiang Gao

**Affiliations:** ^1^ Cancer Virology Program, UPMC Hillman Cancer Center University of Pittsburgh School of Medicine Pittsburgh Pennsylvania USA; ^2^ Department of Microbiology and Molecular Genetics University of Pittsburgh School of Medicine Pittsburgh Pennsylvania USA

**Keywords:** Epstein‐Barr virus, EBV, gammaherpesvirus, GHV, immune evasion, Kaposi's sarcoma‐associated herpesvirus, KSHV, latency and reactivation, model systems, murine gammaherpesvirus 68, MHV68, pathogenesis

## Abstract

Epstein‐Barr virus (EBV) and Kaposi's sarcoma‐associated herpesvirus (KSHV) are oncogenic human gammaherpesviruses (GHVs) associated with a broad spectrum of malignancies and chronic diseases. However, direct studies of these viruses in humans are limited by ethical constraints, technical challenges, and their strict species specificity. To overcome these barriers, researchers have developed surrogate models, with murine gammaherpesvirus 68 (MHV68) emerging as a tractable and widely utilized system. MHV68 shares key genetic and biological features with EBV and KSHV, providing critical insights into GHV pathogenesis, including viral latency, reactivation, immune evasion, and virus‐host interactions. This review summarizes current cellular and animal models for GHV research, highlighting the advantages and limitations of MHV68 as a model for studying human GHVs. We explore mechanisms of viral gene function, immune modulation, and host responses, and discuss how these models have advanced our understanding of GHV‐associated diseases. Improved model systems will be essential for guiding future experimental approaches and developing targeted therapies for GHV‐driven malignancies and other related disorders.

**Abbreviations**
AGS: a cell line isolated from a gastric adenocarcinoma
AIDS: acquired immuno‐deficiency syndrome
Akata: a cell line isolated from a Burkitt lymphoma
ALI: air liquid interface
APC: antigen presenting cells
BAC: bacterial artificial chromosome
BAC‐RGB: bacterial artificial chromosome harboring red, green and blue fluorescent protein cassettes
BAL: broncho‐alveolar lavage
BCBL‐1: KSHV+/EBV− primary effusion (body cavity‐based) lymphoma cell line
BCP‐1: KSHV+/EBV− primary effusion (body cavity‐based) lymphoma cell line isolated from peripheral blood mononuclear cells
BCR: B cell receptor
BC‐1: a KSHV+/EBV+ primary effusion (body cavity‐based) lymphoma cell line
BC‐3: a KSHV+/EBV− primary effusion (body cavity‐based) lymphoma cell line
BEC: blood vessel endothelial cell
BL: Burkitt lymphoma
BRG: Balb/c Rag2^−/−^ Il2rg^−/−^
DLBCL: diffuse large B cell lymphoma
EBERs: Epstein‐Barr virus‐encoded small RNAs
EBNAs: Epstein‐Barr nuclear antigens
EBV: Epstein‐Barr virus
ECFC: endothelial colony forming cells
ECM: extracellular matrix
FDC: follicular dendritic cells
GC: gastric carcinoma
GHV: gammaherpesvirus
GPCR: G protein‐coupled receptor
HIV: human immunodeficiency virus
HL: Hodgkin lymphoma
HLH: hemophagocytic lymphohistiocytosis
hMSCs: human mesenchymal stem cells
HSCs: hematopoietic stem cells
hTERT: human telomerase reverse transcriptase
HUVEC: human umbilical vein endothelial cells
IL‐6: Interleukin‐6
IM: infectious mononucleosis
KICS: Kaposi's sarcoma inflammatory cytokine syndrome
KS: Kaposi's sarcoma
KSHV: Kaposi's sarcoma‐associated herpesvirus
LANA: latency‐associated nuclear antigen
LEC: lymphatic endothelial cells
lncRNAs: long non‐coding RNAs
LPD: lymphoproliferative disorder
MCD: multicentric Castleman's disease
MCMV: Murine cytomegalovirus
MHC: major histocompatibility complex
MHV68: Murine gammaherpesvirus‐68
MSC: mesenchymal stem cells
MSM: men who have sex with men
Mutu: an EBV‐positive cell line isolated from a Burkitt lymphoma
NHL: non‐Hodgkin lymphoma
NHP: non‐human primate
NOD: non‐obese diabetes
NPC: nasopharyngeal carcinoma
NSG: NOD/LtSz‐scid Il2rg^−/−^
ORF: open‐reading‐frame
PBMC: peripheral blood mononuclear cells
PDX: patient derived xenograft
PD‐L1: programmed death ligand 1
PEL: primary effusion lymphoma
PRR: pattern recognition receptors
PTLD: post‐transplant lymphoproliferative disorder
Raji: an EBV‐positive cell line isolated from a Burkitt lymphoma
RF: retroperitoneal fibromatosis
RFHV: retroperitoneal fibromatosis‐associated herpesvirus
rhLCV: Rhesus lymphocryptovirus
RRV: Rhesus monkey rhadinovirus
RTA: replication and transcriptional activator
SCID: severe‐combined immunodeficiency syndrome
SIV: Simian immunodeficiency virus
SLE: systemic lupus erythematosus
Tfh: follicular T helper cells
TIME: telomerase‐immortalized human microvascular endothelial cells
TIVE: telomerase‐immortalized human umbilical vein endothelial cells
TLRs: Toll‐like receptors
Treg: regulatory T cells
VCA: viral capsid antigen
XLP: X‐linked lymphoproliferative disease
ZTA: EBV BZLF1 or ZEBRA

## 1 Introduction

Gammaherpesviruses (GHVs) belong to a subfamily of *Herpesviridae*, comprising large, enveloped double‐stranded DNA (dsDNA) viruses that establish lifelong latency in host cells. Among the most clinically significant members of this subfamily are the two human GHVs: EBV and KSHV [1]. EBV and KSHV are classified as DNA tumor viruses due to their association with various human cancers [1]. Despite their differences in tissue tropism and gene regulation, EBV and KSHV share key biological features: lifelong persistence and latency, evasion of host immunity, genomic homology, the capacity to activate oncogenic signaling pathways, and induction of human cancers [2, 3]. Additionally, like all herpesviruses, they exhibit a biphasic life cycle, alternating between lytic replication and latency [4, 5]. The balance between lytic replication and latency is a key determinant of viral pathogenesis and oncogenicity. Understanding the similarities and differences between EBV and KSHV provides critical insights into GHV biology and viral tumorigenesis.

This review evaluates the model systems used to study EBV and KSHV, with a focus on their molecular biology, immune response, pathogenesis, and translational applicability. We begin with an overview of their natural history, life cycle, immune evasion, virus‐host interactions, and associated diseases. We then discuss in vitro and in vivo model systems, with a particular emphasis on MHV68 as a tractable surrogate model for EBV and KSHV. Finally, we assess the relevance of these systems for understanding tumorigenesis and immune evasion; and critically evaluate the strengths and limitations of MHV68 as a model for human GHVs. A comprehensive understanding of these aspects is essential for translating laboratory and preclinical findings into therapeutic strategies for GHV‐associated diseases.

## 2 History and Discovery

The concept of viral oncogenesis was revolutionized in 1911 when Peyton Rous discovered the first oncogenic virus in chickens, laying the foundation for modern tumor virology [6]. However, it was not until 1964, more than five decades later, that the first human oncogenic virus was identified [7]. In 1958, Irish surgeon Denis P. Burkitt [8] described a pediatric lymphoma (Burkitt lymphoma, BL) that affected a large population of children in sub‐Saharan Africa. Subsequent investigations by Anthony Epstein, Yvonne Barr, and Bert Achong led to the discovery of virions within BL cancer cells, culminating in the identification of EBV as the etiological agent of BL [9]. Seroprevalence is high (> 95%) among adults worldwide, indicating chronic infection in a vast population [10]. EBV has been associated with a range of epithelial cell and B‐cell malignancies including nasopharyngeal carcinoma (NPC) [11], a subset of gastric carcinoma (GC) [12], non‐Hodgkin lymphoma (NHL) [13], Hodgkin lymphoma (HL) [14], and others, in addition to causing infectious mononucleosis (IM) [15].

Thirty years after the discovery of EBV, another landmark discovery was made with the identification of KSHV, also known as human herpesvirus 8 (HHV‐8). First described by Moritz Kaposi in the 1870s, Kaposi's sarcoma (KS) was considered to be a blood vessel tumor primarily seen in Mediterranean and Eastern European men [16]. In the 1960s, endemic KS was observed in sub‐Saharan Africans [17], and in the early 1980s, KS reemerged in epidemic form among men who have sex with men (MSM), particularly in those infected with HIV [18, 19, 20]. Cases in women were often traced to bisexual male partners prompting the hypothesis that KS was caused by an as‐yet‐undiscovered infectious agent [21]. In 1994, Yuan Chang and Patrick Moore identified novel herpesvirus DNA sequences in KS lesions, leading to the discovery of KSHV as the causative agent [22]. Since then, KSHV has also been associated with primary effusion lymphoma (PEL) [22, 23], some forms of multicentric Castleman's disease (MCD) [24], and KSHV‐associated inflammatory cytokine syndrome (KICS) [25], further highlighting its oncogenic and immunomodulatory potential. Unlike EBV, the seroprevalence of KSHV in the general population is low in North America and Europe, but it is in the median range in Mediterranean and Eastern European regions, and high in sub‐Saharan Africans [26, 27, 28, 29]. Both EBV and KSHV can cause malignancy in immune‐competent individuals, but at elevated incidence in the immune‐compromised [2, 3].

## 3 Gammaherpesvirus Biology

### 3.1 Acute Infection and Lytic Replication

**Figure 1:**
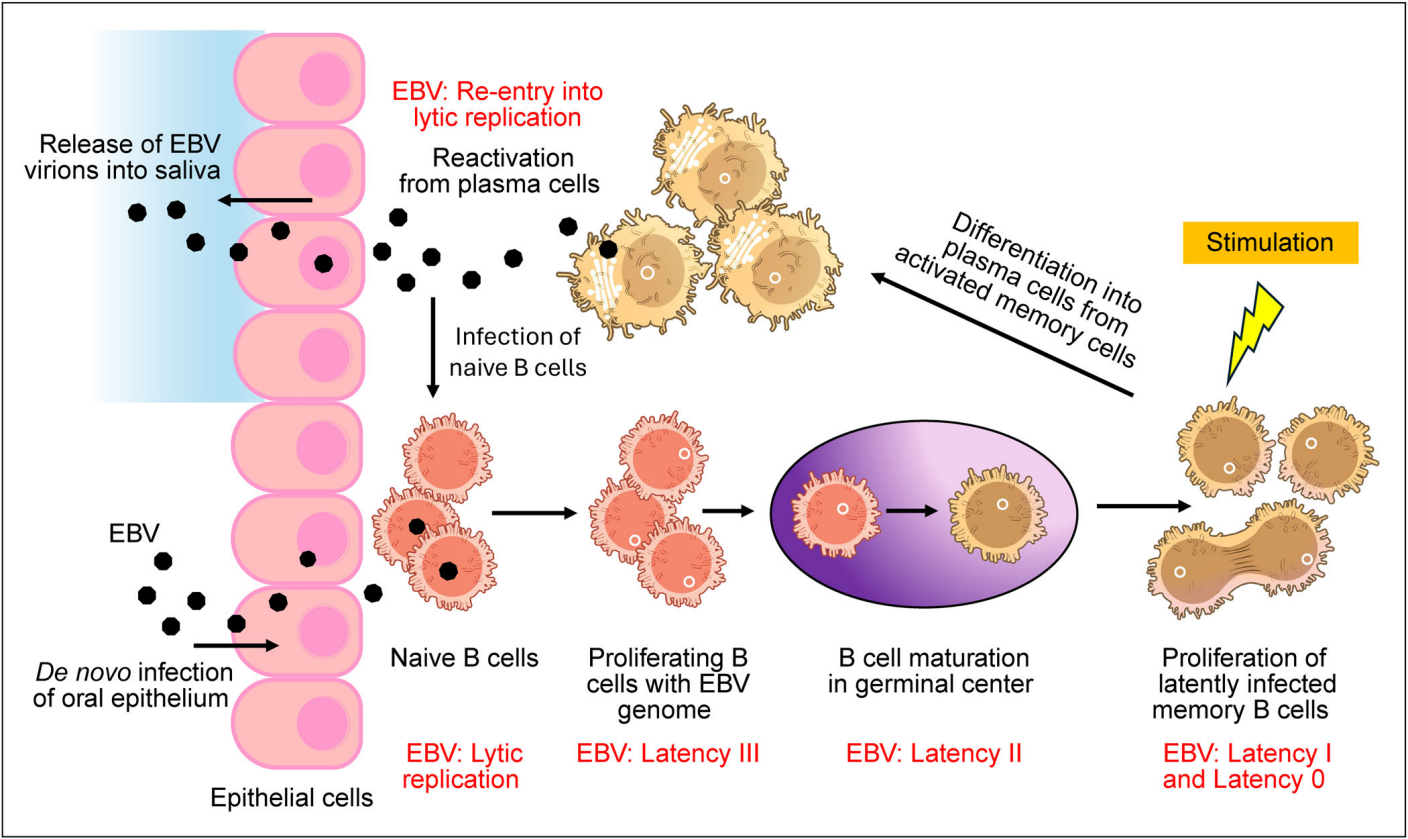
An illustrative model of the life cycle of the Epstein‐Barr Virus. The virus infects and undergoes lytic replication in the oral epithelium and resident B‐cells. B lymphocytes serve as the primary reservoir of the virus, with various stages of B‐cell maturation corresponding to different latency programs. The virus can reactivate and re‐enter lytic phase in plasma cells, thus releasing progeny virions which can be excreted in the saliva. This figure was created on Adobe Illustrator with icons sourced from the National Institute of Allergy and Infectious Diseases, National Institute of Health Bioart Source.

EBV is primarily transmitted through oral contact and the virus initially infects oral epithelial cells or infiltrating lymphocytes, initiating a lytic replication cycle characterized by a temporally regulated cascade of gene expression [30]. This process begins with the expression of immediate‐early (IE) genes, BZLF1 and BRLF1, encoding the transcriptional activators ZTA and RTA, respectively [31]. These proteins, in turn, activate the expression of early (E) genes involved in viral DNA replication. Once replication is underway, the late (L) genes encoding structural components of the virion are expressed, culminating in the assembly and release of progeny virions. Lytic replication causes tissue damage and provokes innate immune responses that eventually limit viral production. Oral hairy leukoplakia is the only known lytic condition that manifests as clinical disease, which in HIV individuals is an indicator of progression to AIDS, that can be treated but not cured by the lytic targeting agent ganciclovir [32]. However, EBV is not cleared from the host; instead, it establishes lifelong latency in B cells via tightly regulated latency programs, which we will discuss in more details in the next section [33, 34] (Figure 1).

Despite a high prevalence, most EBV infections remain asymptomatic or cause mild illness [35]. In adolescents and young adults, primary infection is usually asymptomatic or sub‐clinical, or presents as IM, an inflammatory condition characterized by lymphadenopathy, sore throat, fatigue, and lymphocytosis [36]. The incubation period typically spans several weeks, during which the host mounts a dynamic and evolving immune response with a gradual switch from “early antibodies” against IE and other lytic proteins to “late antibodies” against latency‐related proteins, reflecting the transition from a primary to a chronic phase of infection [37]. Notably, during acute infection, the host develops IgM and IgG antibodies against viral capsid antigens (VCA), peaking within 6 weeks [38]. In contrast, antibodies against Epstein‐Barr nuclear antigens (EBNAs), which mark the shift to viral latency and persistent infection, emerge later [37]. We further discuss the immune response against EBV infections in a later section.

KSHV, like EBV, is also primarily acquired via oral transmission and initially infects epithelial, endothelial, and B cells in the oropharynx and tonsils [39, 40, 41]. However, unlike EBV, KSHV primary infection is almost always asymptomatic, and its clinical manifestations are more dependent on host immunocompetence. Upon de novo infection, KSHV initiates lytic replication, with the ORF50 gene product RTA, the master transcriptional activator, being both necessary and sufficient for lytic gene expression and lytic replication [42]. As with EBV, gene expression proceeds in a temporal cascade: IE genes encoding transcription factors are expressed first, followed by E genes involved in DNA replication, and then L genes encoding structural proteins, culminating in the generation of infectious virions [43].

After an initial burst of lytic activity, KSHV switches to latency [44, 45]. Upon entry into the cells, the viral genome is epigenetically modified [46]. The initial RTA expression induces chromatin remodeling and the deposition of activating histone marks like H3K27ac and H3K4me3 at various loci on the viral genome, including the promoter of the ORF73 encoding latency‐associated nuclear antigen (LANA) [46]. As the infection progresses, LANA represses RTA expression by recruiting the polycomb repressive complex, thereby silencing lytic genes and promoting latency [46]. Whether lytic replication continues or is curtailed depends on the cell type and microenvironmental cues. In most settings, KSHV quickly establishes latency, a state that is often non‐cytolytic and may even enhance cell survival [47]. Early during infection, KSHV activates pro‐survival pathways such as Akt/mTOR/PI3K, thereby priming cells for cellular transformation [48]. Lytic replication facilitates the spread of the virus to new cells and enables the expression of viral homologs of cytokines and products that induce inflammatory and angiogenic factors, such as viral G protein‐coupled receptor (vGPCR) encoded by ORF74, viral interleukin‐6 (vIL‐6) encoded by ORF‐K2 and viral miRNAs [49, 50, 51]. These viral products create a positive feedback loop that contributes to the rapid dissemination and development of early stages of KS tumors. The serendipitous finding that treatment of HIV patients with ganciclovir reduced KS incidence by up to 93% further support the claim that lytic replication contributes to the risk of developing KS [52].

### 3.2 Viral Latency and Persistence

Following primary infection and an initial wave of lytic replication, both EBV and KSHV establish latency. This transition allows the virus to evade immune surveillance and maintain a stable reservoir of infected cells, ensuring lifelong persistence [5, 34]. Latency is not merely a dormant state; it is a dynamic, highly regulated phase in which the viral genome persists as an episome in the host nucleus and expresses a limited subset of viral genes that promote cell survival, inhibit apoptosis, and modulate immune recognition.

In EBV infections, B lymphocytes serve as the primary latency reservoir. Viral entry into B cells is mediated by binding to CD21 (complement receptor 2) and HLA Class II molecules [53], leading to internalization of the virus and initiation of the latency program. EBV latency is classified into four distinct programs: Latency 0, I, II, and III, defined by distinct expression patterns of latent genes, which reflect both, the immune microenvironment and the stage of B cell differentiation (Figure 1) [34]. Latency III, the most transcriptionally active form, features expression of all six Epstein‐Barr nuclear antigens (EBNA1, EBNA2, EBNA3A, EBNA3B, EBNA3C, and EBNA‐LP), latent membrane proteins (LMP1, LMP2A, LMP2B), and non‐coding RNAs such as EBERs and miRNAs. This program is typically seen in lymphoblastoid cell lines and in post‐transplant lymphoproliferative disorders (PTLD). Latency II, observed in HL and NPC, involves the expression of EBNA1, LMP1, and LMP2A/B, EBERs, and miRNAs. Latency I, characteristic of BL, in which the expression of viral genes is restricted to EBNA1 and non‐coding RNAs, while Latency 0 is found in resting memory B cells and features minimal viral gene expression, ensuring long‐term persistence and immune evasion.

The switch between different latency programs corresponds to different stages of B cell development and is a heavily studied area in EBV research. Latency allows EBV to evade host immune responses by encoding numerous immune evasion proteins in addition to limiting antigen expression. EBNA1, for example, evades proteasomal degradation due to its glycine‐alanine repeat (GAR) domain, thereby limiting MHC Class I presentation [54], while LMP1 mimics a constitutively active CD40 receptor, activating NF‐κB and JAK/STAT pathways among others which promote B cell survival and proliferation [55]. This tightly controlled balance between latent gene expression and immune modulation predicates EBV's capacity for persistence and its oncogenic potential.

**Figure 2:**
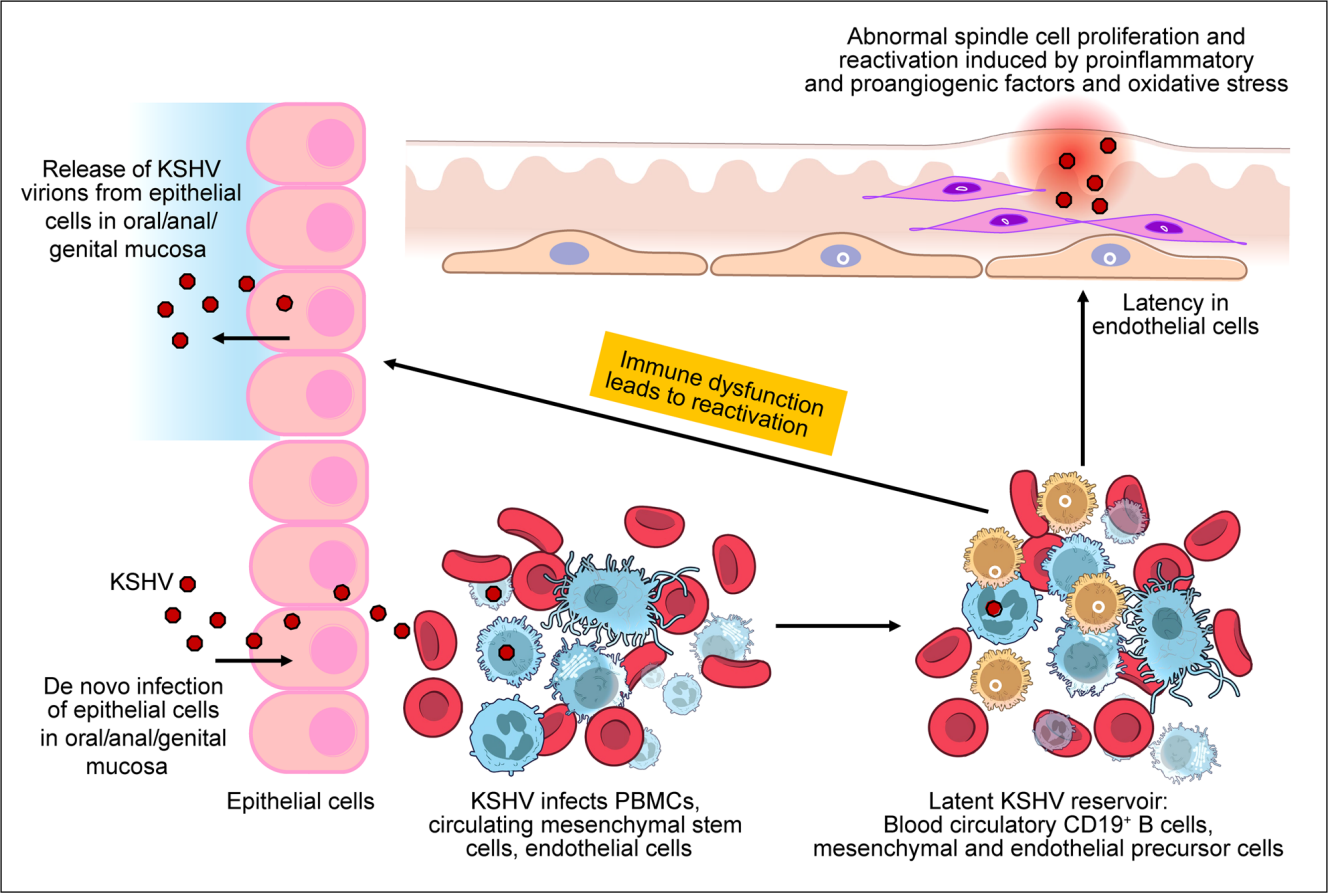
An illustrative model of the life cycle of the Kaposi sarcoma‐associated herpesvirus. The virus enters through and infects the epithelial cells of the oral/anal/genital mucosa. Thereafter it is spread to circulating PBMCs, mesenchymal stem cells, precursor cells and endothelial cells. The virus eventually establishes latency in B cells, mesenchymal and endothelial precursor cells, and endothelial cells. Immune dysfunction leads to reactivation of the virus. In endothelial cells, in the presence of pro‐inflammatory and pro‐angiogenic signals, the virus can lead to formation of KS lesions. Reactivated virus is also shed from epithelial cells in the oral, anal, or genital mucosa. This figure was created on Adobe Illustrator with icons sourced from the National Institute of Allergy and Infectious Diseases, National Institute of Health Bioart Source.

KSHV also establishes latency shortly after initial infection, following an early burst of lytic gene expression [44, 45] (Figure 2). Although KSHV can infect multiple cell types, including B cells, endothelial cells, and monocytes, the extent to which endothelial cells contribute to the latency reservoir in the absence of KS remains unclear [39]. Nevertheless, establishment of latency and long‐term maintenance in culture have been demonstrated for endothelial cells like blood vessel endothelial cells (BECs), lymphatic endothelial cells (LECs) and human umbilical vein endothelial cells (HUVECs), and mesenchymal stem cells (MSCs) [44, 56, 57, 58]. These findings raise the possibility that KSHV‐infected individuals may harbor viral latent genomes in endothelial cells and precursor cells even in the absence of any KSHV‐associated diseases.

In immunocompetent individuals, KSHV predominantly adopts a latent transcriptional profile. KSHV latency is maintained by a limited set of viral gene products including LANA, vFLIP (ORF72), vCyclin (ORF71), Kaposins (ORF‐K12), miRNAs, and small nuclear RNAs [59, 60, 61, 62, 63, 64]. These factors work cooperatively to promote cell survival, proliferation, and immune evasion [3]. vFLIP activates NF‐κB signaling and inhibits apoptosis, while vCyclin dysregulates the cell cycle by activating CDKs and bypassing normal cell cycle checkpoints [65, 66]. KSHV‐encoded miRNAs further enhance latency by promoting cell proliferation and survival, and targeting both viral and cellular transcripts involved in immune recognition and cell stress responses [50, 67, 68, 69, 70].

KSHV latency establishment and maintenance is a multifactor process involving many steps that are yet to be completely elucidated. LANA mediates latent genome replication and tethers the viral episome to the host chromosomes, thus ensuring persistence of viral genomes in the host cell and equitable distribution of the episomes into daughter cells during cell division [71]. LANA also promotes cell proliferation and survival, and epigenetically inhibits viral lytic gene expression [72]. In vitro studies have shown that LANA regulates epigenetic modifications including histone acetylation and methylation of the viral episome [73]. Importantly, KSHV latency is not static. Environmental stressors, co‐infections, inflammatory cytokines, and hypoxia can relieve repression of lytic promoters and reactivate the lytic cycle, contributing to viral dissemination and disease progression [74, 75]. In immunocompromised individuals, such as HIV‐positive patients or organ transplant recipients, loss of immune control often leads to increased lytic replication and progression to KSHV‐associated malignancies.

The strategies employed by EBV and KSHV to establish and maintain latency reflect their co‐evolution with the human immune system, exemplified by the higher diversity of genetic variants in latent genes [76, 77, 78]. By selectively expressing viral genes and manipulating host signaling pathways, these viruses evade immune detection while maintaining capacity for reactivation. A thorough understanding of latency‐associated mechanism is thus central to therapeutic efforts aimed at targeting latent reservoirs or inducing controlled reactivation for virus‐directed cytotoxic strategies [79, 80, 81, 82].

### 3.3 Reactivation

The ability of EBV to toggle between latent and lytic phases is critical for its lifelong persistence in memory B cells and is closely tied to its oncogenic potential [33]. Because the EBV genome is largely epigenetically silenced during latency, reactivation requires coordinated molecular events to overcome this transcriptional repression [4]. Lytic reactivation begins with the induction of IE genes. Physiological stimuli such as B cell receptor (BCR) activation, epithelial differentiation, and various cellular stress signals like caspase and inflammasome activation can initiate this process [83, 84, 85, 86]. These signals activate the viral Zp and Rp promoters, driving the expression of IE genes BZLF1 and BRLF1, respectively, which together orchestrate the subsequent lytic gene expression cascade and initiate productive viral replication [87, 88]. Reactivation is further modulated by a variety of host transcription factors. Positive regulators such as C/EBP‐beta, X‐box binding protein 1 (XBP1) and hypoxia‐inducible factor 1‐alpha (HIF‐1α) enhance BZLF1 expression and promote lytic gene expression [89, 90, 91]. Other factors like PIAS1, YTHDF2, SAMHD1, MYC, STAT3, and TRIM28 act as negative regulators whose depletion facilitates reactivation [84, 92, 93, 94, 95, 96, 97, 98]. Some of these regulators, such as DDX5 and DDX17, are common pro‐viral factors for EBV and KSHV [99]. The complex co‐ordination of the lytic cascades involves disruption of epigenetics and chromatin remodeling [100, 101]. This intricate interplay between viral and host factors enables EBV to adaptively respond to microenvironmental cues, optimizing its persistence and oncogenic capacity under different physiological and pathological conditions.

Similarly, KSHV reactivation is critical not only for viral propagation but also for tumorigenesis, particularly in the early stages of KS. Although most KSHV‐infected tumor cells are latently infected, expression of lytic genes contributes to disease progression by inducing angiogenic, mitogenic, and pro‐inflammatory signaling pathways [102, 103, 104, 105, 106]. Moreover, reactivation enables de novo infection of neighboring uninfected cells, and induces angiogenic and pro‐inflammatory signaling pathways through ligand‐receptor interactions, and further expands the population of latently infected cells with oncogenic potential [107, 108, 109].

KSHV reactivation is induced by a broad array of physiological and environmental triggers, including oxidative stress, hypoxia, inflammation, metabolic dysregulation (e.g., high glucose), co‐infections, apoptotic signals, and immune suppression [74, 75]. The central molecular driver of lytic reactivation is RTA that initiates the lytic cascade [42]. In addition, multiple studies have highlighted the role of viral miRNA and long non‐coding RNAs (lncRNAs) in regulating this switch [50, 110, 111, 112, 113, 114]. During latency, LANA represses lytic gene expression and tethers the viral episome to the host chromosome by forming a complex with the chromatin remodeler CHD4 [115]. Upon reactivation, KSHV lncRNAs disrupt this complex by sequestering CHD4, thereby detaching the viral episome from the host genome and enabling the initiation of viral DNA replication. Concurrently, epigenetic reprogramming occurs, with epigenetic repressive marks being replaced by active marks, facilitating lytic gene expression [114, 115].

The precise control of latency and lytic replication is essential for viral persistence and host adaptation. However, dysregulation of these processes, particularly under conditions of immune suppression, chronic inflammation, or metabolic stress, can shift the balance toward pathological reactivation [74, 75]. Such disruption plays a pivotal role in viral oncogenesis, a topic that will be explored in the subsequent sections.

## 4 Gammaherpesvirus Associated Diseases, Their Transmission, and Prevalence

Globally, EBV and KSHV are responsible for over 200 000 new cancer cases annually, posing a significant health burden due to their high prevalence and association with lymphoproliferative disorders (LPDs) and malignancies [116, 117]. This burden is especially pronounced in low‐ and middle‐income countries where both viruses are prevalent and healthcare infrastructure is limited [118].

### 4.1 Epidemiology of the Epstein‐Barr Virus

Despite their widespread distribution, particularly EBV, which infects nearly all adults, GHV‐associated diseases usually require co‐factors for progression from asymptomatic infection to severe illness or malignancy [119, 120]. EBV is primarily transmitted via saliva through activities such as kissing and sharing utensils, drinks, or toothbrushes [121]. During active lytic infection, EBV is shed in the saliva and can continue to be shed intermittently throughout a person's life, regardless of symptoms [121]. Shedding in breast milk has been documented and although rare, perinatal transmission has been suspected [122, 123]. In early childhood, EBV infection is typically asymptomatic, facilitating undetected spread [37]. Symptomatic infections such as IM are more common in adolescents and adults [121]. Social environments such as schools, daycares, and college dormitories are major hubs for transmission. In lower‐income regions, children typically acquire EBV early in life through close contact with caregivers [37], while in higher‐income regions, infections tend to occur later, during adolescence or early adulthood [36, 121]. Although EBV maintains lifelong latency and adult seroprevalence exceeds 90%, clinical disease remains relatively rare. Reactivation, however, can lead to chronic active infection characterized by prolonged fever, lymphadenopathy, hepatosplenomegaly, and elevated liver enzymes. In severe cases, reactivation can trigger hemophagocytic lymphohistiocytosis (HLH), a potentially fatal inflammatory syndrome [124].

EBV's oncogenic potential is among its most concerning clinical features. It is etiologically linked to several human cancers. EBV drives endemic BL (eBL) in malaria‐endemic regions by activating c‐MYC which leads to uncontrolled proliferation of affected B cells [93, 125]. It is almost universally detected in all histological subtypes of NPC prevalent in Southeast Asia, where latent viral proteins like LMP1 drive epithelial cell transformation [11, 126]. EBV is also implicated in a subset of GCs where latent proteins disrupt apoptosis and promote proliferation [127]. Emerging research also suggests a role for EBV in autoimmune diseases such as rheumatoid arthritis, multiple sclerosis, and systemic lupus erythematosus (SLE) [128, 129, 130]. SLE patients, for instance, often show 10‐fold higher EBV loads in peripheral blood mononuclear cells (PBMCs) compared to healthy individuals [131, 132]. Auto‐antibodies that cross‐react with EBNA1 could explain a subset of patients with multiple sclerosis [130, 133].

EBV‐associated LPDs are driven by uncontrolled B cell proliferation. A severe form of LPD called PTLD arises in immunosuppressed individuals following organ transplantation, most frequently from solid organ transplant, and pediatric transplant recipients being at the highest risk [134, 135]. Due to the loss of T cell‐mediated control in transplant recipients on immunosuppressive therapy, PTLD can range from benign polyclonal B cell hyperplasia to aggressive lymphoma [136, 137]. However, PTLD in HSCT patients is relatively rare nowadays. CD19^+^ B‐cell depletion with or without autologous CTL infusion is used to prevent EBV reactivation and PTLD [138]. EBV also contributes to the pathology of a rare disorder called X‐linked lymphoproliferative syndrome (XLP), where infection results in uncontrolled immune activation, liver failure, HLH, and B‐cell malignancies [139]. The virus is further implicated in a subset of HLs and certain NHLs, particularly in immunocompromised individuals largely through the virus's ability to modulate B cell signaling pathways and cell survival pathways [140, 141].

EBV‐driven oncogenesis is strongly influenced by co‐infections with co‐endemic agents, especially in sub‐Saharan Africa [120, 142, 143]. A notable example is the synergistic interaction between EBV and *Plasmodium falciparum*, the causative agent of malaria, in the development of eBL. The hallmark of BL is the c‐MYC/IgH translocation leading to uncontrolled B cell proliferation [125]. Chronic malaria contributes to eBL by inducing polyclonal B‐cell activation, increasing the likelihood of EBV‐infected B‐cell expansion and subsequent genetic errors, including c‐MYC translocations [120, 144]. Additionally, chronic inflammation from malaria infection causes T cell exhaustion, leading to increased EBV loads and impaired immune responses, allowing EBV‐infected B cells harboring oncogenic mutations to evade immune clearance [145, 146].

### 4.2 Epidemiology of the Kaposi Sarcoma‐Associated Herpesvirus

KSHV is primarily transmitted through the oral or anogenital mucosa, often via sexual contact, which explains the elevated KS rates among MSM, with seroprevalence ranging from 25% to 60%, far exceeding the < 10% in the general population in non‐endemic regions [116, 147]. Heterosexual transmission is less common, and women generally have lower infection rates [148].

HIV co‐infection worsens the burden of both EBV and KSHV. Immunosuppression due to HIV increases susceptibility to GHV reactivation and the risk of virus‐driven malignancies. HIV and GHV co‐infections are particularly prevalent in sub‐Saharan Africa, where all three viruses are endemic [119, 148, 149]. The HIV epidemic has driven a surge in KS incidence, especially in East and Southern Africa, making KS the most common HIV‐associated cancer in countries like Uganda, Malawi, and Zimbabwe [150, 151, 152, 153]. KSHV seroprevalence is significantly higher in HIV‐positive populations compared to HIV‐negative groups in sub‐Saharan Africa [27, 148, 149]. Although the advent of antiretroviral therapy (ART) has reduced KS incidence in HIV‐positive individuals [152, 154], late diagnosis and limited healthcare access continue to make KS a leading cause of cancer‐related morbidity and mortality in these populations [118].

KSHV is also responsible for endemic KS, which occurs independently of HIV in sub‐Saharan Africa, as well as classical KS, a slow growing malignancy typically seen in elderly Mediterranean populations [27]. These, along with Iatrogenic KS, which arises in organ transplant recipients on immunosuppressive therapy indicate that KSHV infection is necessary but not sufficient for KS development, underscoring the importance of cofactors such as immunosuppression, HIV‐encoded proteins, immune senescence, chronic inflammation, co‐infections, and even recreational drug use [151, 155, 156, 157].

Endemic KS is prevalent in certain regions including Uganda, Kenya, Tanzania, Zambia, South Africa, with higher rates in areas of East and Central Africa [27, 152, 153]. This is consistent with the high KSHV seroprevalence in these areas ranging from 20% to 50%, compared to < 10% in many Western countries [151, 156]. Epidemiological evidence supports horizontal transmission via household contact, as KSHV DNA has been found in maternal saliva and breast milk [158]. Familial clustering is also observed [159]. Children as young as 1 or 2 years old are affected, often due to immunosuppression from chronic infections (e.g., malaria, tuberculosis, schistosomiasis) or malnutrition [151]. Immune dysregulation due to chronic antigenic stimulation probably enhances KSHV reactivation and tumorigenesis. However, endemic KS is often underreported or misclassified due to the overwhelming burden of AIDS‐KS in these populations [156, 157, 160]. Children with endemic KS may also present with HIV co‐infection due to the overlap of risk factors [151].

Classical KS, predominantly seen in Mediterranean, Eastern European, some Arabic populations, and China's Xinjiang province is typically a slowly developing disease of older men. The median KSHV seroprevalence in these regions correlates with KS incidence, with prevalence in the general population ranging from 4% to 24% [27, 161]. The specific drivers of elevated seroprevalence and transmission modes in these regions remain poorly understood.

Iatrogenic KS occurs in transplant recipients, reflecting the role of immunosuppressive therapy in reactivating latent KSHV and loss of control on proliferating tumor cells. Calcineurin inhibitors, in particular, promote viral replication and angiogenesis, facilitating tumor growth [155, 162]. However, direct transmission of KSHV from organ donors to recipients have been documented [163]. While the incidence of iatrogenic KS is lower than that of AIDS‐KS or endemic KS, it remains clinically significant due to the need to balance antirejection therapy with oncologic management. Differences in socio‐economic status, education levels, age, and immune‐related genes may influence KS susceptibility, including the Uyghur, Kazakh, and Han populations in China's Xinjiang province [159, 161, 164].

KSHV is also implicated in all HIV‐associated and some HIV‐negative MCD, a rare LPD that causes systemic symptoms such as fever, weight loss, and lymphadenopathy driven by excessive production of viral and cellular interleukin‐6 (IL‐6), leading to immune dysregulation and proliferation of B cells [165]. PEL, a KSHV‐associated lymphoma, typically occurs in body cavities of HIV‐positive patients [40]. EBV co‐infection is common in PEL and may stabilize KSHV infection and promote tumorigenesis, as demonstrated in humanized mouse models [166].

In summary, the global distribution and burden of EBV and KSHV‐associated diseases reflect a complex interplay between viral persistence, immune status, co‐infections, and environmental or genetic factors. Both viruses exploit immunosuppressive states, whether induced by HIV infection, immunosuppression during organ transplantation, chronic co‐infections, or age‐related immune decline, to reactivate and drive oncogenesis. Despite medical advances, these viruses continue to pose a serious threat to public health in resource‐limited settings.

While a hallmark of human GHVs is their restricted host range, there are many economically important GHVs that infect agricultural animals. Bovine GHV infection in cows can cause endometritis, vulvovaginitis, and mastitis [167, 168]. Ovine herpesvirus 2 and Alcelaphine herpesvirus 1 and 2 cause malignant catarrhal fever (MCF), a disease primarily affecting cattle in Africa [169, 170, 171]. These infections can have devastating effects on economies where cattle farming plays an important role. Additionally, GHVs are also known to cause disease in cats, elephants, and whales [172, 173, 174]. While this topic is outside the scope of this review, these are important fields of study due to their impacts on the well‐being of the animals who share the world with us.

## 5 Immune Response and Host‐Virus Interactions in Human GHV Infection

### 5.1 Innate and Adaptive Immune Responses

#### 5.1.1 Immune Response to EBV

EBV infection activates a broad spectrum of innate immune sensors, including multiple pattern recognition receptors (PRRs) such as Toll‐like receptors (TLRs) [175, 176]. These sensors initiate signaling cascades that culminate in robust immune effector responses. During acute infection, EBV induces a pronounced pro‐inflammatory cytokine milieu, characterized by elevated levels of Type I interferons (IFN‐α/β), IL‐6, TNF‐α, and IFN‐γ, cytokines that contribute to the clinical manifestations of IM [177].

EBV products are detected by distinct TLRs in various immune cells. TLR3 recognizes double‐stranded RNA motifs, such as EBERs [178]. Elevated serum EBERs possibly released from dying cells, are observed in IM, chronic active EBV infection, and EBV‐associated HLH, conditions marked by excessive cytokine production [179]. These circulating EBERs activate TLR3‐dependent cytokine secretion and dendritic cell (DC) maturation, suggesting a key role for EBER‐TLR3 interactions in initiating EBV‐specific T cell responses [177].

Similarly, EBV genomic DNA, which is unmethylated within virions but becomes methylated post‐infection [180], is recognized by TLR9 in plasmacytoid DCs (pDCs) [175]. This receptor targets unmethylated CpG motifs, making cell‐free EBV DNA a potent activator. Numerous EBV antigens are also recognized by TLR3 and TLR9 on DCs [175, 181]. In humans, TLR3 and TLR9 are localized to distinct DC subsets, TLR3 to classical DCs (cDCs) and TLR9 to pDCs, enabling these cells to recognize different EBV‐associated cues [182]. However, EBV BGLF5 downregulates TLR9 signaling to counteract this immune detection during lytic replication [176]. Additionally, monocytes recognize EBV through TLR2, triggering cytokine and chemokine secretion [182]. Hence, TLR‐mediated recognition of EBV activates DCs, forming an initial barrier to infection by producing antiviral Type I interferons (IFN‐α/β) [181]. In a model where PBMCs from humanized SCID mice were supplemented with pDCs, IFN‐α production was shown to be crucial for controlling EBV [183]. Independent studies have consistently shown that TLR9‐dependent IFN‐α production in pDCs plays a critical role in suppressing viral replication and bridging innate and adaptive immune responses [177].

While cytokine storms characterize early immune activation, the adaptive immune response is essential for long‐term EBV control. Signals from innate immune system prime the adaptive arm, particularly cytotoxic CD8^+^ T cells, which are crucial for targeting both lytic and latent antigens. During acute infection, up to 40%–50% of circulating CD8^+^ T cells are specific for EBV lytic proteins such as BZLF1 and BRLF1, contributing to the lymphocytosis observed in IM [184]. Antigen‐presenting cells (APCs), particularly monocyte‐derived DCs, are central to CD8^+^ T cell priming. While pDCs and EBV‐infected B cells have limited roles in this process, monocyte‐derived DCs are activated through TLR2 and TLR3, with TLR3 stimulation significantly enhancing costimulatory molecule expression and antigen presentation capacity [177].

B cells, the primary targets of EBV, also express TLR3 and TLR9, though their antigen‐presenting function is compromised by viral immune evasion strategies, particularly through LMP1, which suppresses TLR9 signaling [185]. DC‐mediated cross‐presentation of EBV‐infected B cell fragments leads to CD8^+^ and CD4^+^ T cell activation [186]. CD4^+^ T cells enhance antiviral immunity by secreting cytokines (e.g., IFN‐γ, IL‐2), aiding CD8^+^ T cell memory formation, and supporting B cell responses [184, 186].

#### 5.1.2 Immune Response to KSHV

The innate immune response to KSHV is similarly multifaceted. KSHV DNA is sensed by IFI16 and cGAS [187, 188, 189, 190], and mRNA by RIG‐I, TLR3, and TLR7 [191, 192, 193], resulting in Type I IFN production and the release of pro‐inflammatory cytokines. In addition, recognition of KSHV envelope glycoproteins by TLR4 activates the NF‐κB pathway and further amplifies the Type I IFN response [194]. Besides triggering a PRR‐mediated innate immune response, KSHV also directly contributes to inflammation through the expression of viral cytokine and chemokine mimics, including vIL‐6, vMIP, and vGPCR [51, 195]. KSHV latently infected cells activate the alternative complement pathway by downregulating the complement regulatory proteins CD55 and CD59, which could induce inflammatory and angiogenic cytokines in addition to activating the STAT3 pathway [196]. KSHV latently infected cells also upregulate TLR4, and coreceptor and adaptor proteins, leading to activation of NF‐κB and STAT3 pathways as well as induction of inflammatory cytokines [197]. This KSHV‐driven inflammatory microenvironment could potentially promote immune cell infiltration, including macrophages, T‐ and B‐ cells, and is a histological hallmark of KS [198, 199]. Therefore, tightly regulated immune responses are essential for preventing KSHV‐induced inflammation and tumorigenesis.

As is evident from the increased KS incidence in individuals with impaired T cell function such as people living with HIV or transplant recipients, T cell‐mediated immunity is central to controlling KSHV infection and preventing KS [61]. For instance, bronchoalveolar lavage (BAL) samples from patients with pulmonary KS have a higher frequency of effector memory CD4^+^ and CD8^+^ T cells compared to blood, though these cells have reduced pro‐inflammatory function [200]. Circulating KSHV‐specific T cells restrict viral reactivation and proliferation of infected cells, while tumor‐infiltrating T cells exert direct cytotoxic effects on KS spindle cells [201, 202]. However, despite their critical roles, KSHV‐specific T cell responses remain poorly characterized. Work by Nalwoga et al. [153] revealed that even in immunocompetent individuals, KSHV‐specific IFN‐γ responses are weak and heterogeneous. In individuals without KS, comprehensive peptide mapping of the entire KSHV proteome showed relatively weak and broad responses, contrasting with the more robust T cell responses elicited by EBV and other herpesviruses [203].

Although direct in vivo evidence for the role of Type I IFNs and CD4^+^ T cells in controlling EBV or KSHV remains limited, studies using MHV68 models have highlighted the importance of CD4^+^ T cells in controlling persistent GHV infection. During MHV68 infection, uninfected myeloid cells present MHV68‐derived peptides via MHC Class II, activating diverse CD4^+^ T helper cells [184]. Similar to murine cytomegalovirus (MCMV), this indirect activation mechanism allows CD4^+^ and CD8^+^ T cells to cooperate in viral control. However, further insights into the regulation of CD4^+^ and CD8^+^ T cell subset by KSHV and EBV is required to improve our understanding of viral persistence and immune‐mediated tissue damage [184].

### 5.2 Immune Evasion by GHVs

The pathogenesis of GHVs, including EBV, KSHV, and MHV68, is tightly linked to their ability to evade host immune surveillance [204, 205, 206]. These viruses have evolved an array of viral proteins and miRNAs that manipulate host cellular pathways, suppress immune recognition, and facilitate lifelong persistence in the host. The immune response to these viruses involves robust humoral and cellular immune responses during both the lytic and latent phases. However, GHVs have evolved sophisticated immune evasion mechanisms, allowing them to persist in the host for life, and highlighting the critical role of host immunity in controlling infection.

#### 5.2.1 EBV Immune Evasion Mechanisms

EBV employs various strategies to evade the host immune response. EBV modulates host cytokine environments to foster immune suppression. EBV disrupts Types I and II IFN responses. Viral proteins such as BFRF1, BGLF4, and gp110 inhibit IFN‐β production by inhibiting IRF3 phosphorylation and blocking key kinase activities [207, 208]. EBV‐encoded miRNAs (e.g., miR‐BART16 and miR‐BART6‐3p) further impair IFN signaling by targeting components like RIG‐I and CBP [209, 210].

Additionally, viral immunoevasins like BNLF2a and vIL‐10, along with EBV‐induced host IL‐10, impair CD4^+^ T cell function, inhibit antigen presentation by APCs, block NK cell cytotoxicity, and promote regulatory T cell (T_reg_) expansion [186, 211]. Chemokines such as CCL17 and CCL22, induced by EBV latent proteins, also enhance T_reg_ recruitment, further dampening immune surveillance [212]. Cytotoxic CD8⁺ T cell responses, which are central to controlling viral infection, are also impaired by EBV by upregulating PD‐L1, driven by EBV latent proteins and viral miRNAs such as miR‐BHRF1‐2 and miR‐BARTs [206, 213, 214, 215]. These effectors enhance PD‐L1 expression via activation of the JAK/STAT and NF‐κB pathways, promoting immune escape and tumor progression [216]. Elevated PD‐L1 expression often correlates with worse prognosis in EBV‐associated malignancies and may exhibit ethnic and tumor‐type variations [206].

EBV also interferes with antigen presentation. T cells express α and β T cell receptor chains that allow them to specifically bind to short peptides presented in the context of either MHC Class I or II [217, 218]. Most host cells express MHC Class I molecules to present antigens to CD8⁺ T cells, while APCs express MHC Class II for CD4⁺ T cell activation [218]. EBV downregulates MHC‐I via proteins like BILF1, limiting CD8⁺ T cell recognition [217]. EBNA1 suppresses its own translation through a GAR domain, limiting its presentation on MHC‐I and evading immune detection [219]. Interestingly, EBV‐associated‐GC exhibits upregulation of MHC‐I and related genes (e.g., TAP1, TAP2, TAPBP), which correlates with increased T and NK cell infiltration, and IFN‐γ expression [206, 220]. In contrast, NPC often shows MHC‐I downregulation, including decreased TAP1 and HLA‐A expression [206, 221]. EBV miR‐BART7 downregulates (MHC Class I polypeptide‐related sequence A) MICA, reducing NK cell‐mediated cytolysis in NPC cells [214, 222]. EBV also significantly impairs MHC‐II expression in B cells. Transcriptomic studies show that EBV downregulates MHC‐II gene expression during B cell immortalization. EBNA2 inhibits the expression of MHC‐II transactivator (CIITA), thereby preventing its enhancer activity, and limiting MHC‐II‐mediated T cell activation [223]. Notably, EBV‐positive diffuse large B cell lymphomas (DLBCLs) exhibit lower MHC‐II expression and more frequent CIITA mutations compared to EBV‐negative cases [215]. These diverse immune evasion strategies collectively contribute to EBV‐driven oncogenesis and immune escape.

#### 5.2.2 KSHV Immune Evasion Mechanisms

KSHV utilizes various strategies to impair the host's innate immune responses. In KSHV‐ infected monocytes, TLR3 expression and its downstream targets, including IFN‐β, CCL2, and CXCL10, are initially upregulated [191]. However, KSHV viral interferon regulatory factors (vIRFs) counteract this response. The KSHV RTA protein facilitates degradation of TRIF, the adaptor for TLR3, and suppresses TLR4 signaling by destabilizing MyD88 mRNA [224]. In endothelial cells, TLR4 signaling is further inhibited via vGPCR‐ and vIRF1‐mediated activation of ERK signaling [225]. KSHV also targets other PRRs. ORF63 binds NLR proteins such as NLRP1, inhibiting inflammasome formation [226]. KSHV ORF64 suppresses RIG‐I by inhibiting its ubiquitination, activation, and downstream antiviral signaling [227]. KSHV ORF52 inhibits cGAS enzymatic activity interfering with cytosolic DNA sensing [189], while LANA and vIRF1 disrupt cGAS‐STING pathway components [188, 190]. Moreover, vIRF1‐4 mimic cellular IRFs, blocking IRF function and suppressing IFN production [228, 229].

Additionally, KSHV impairs the adaptive immune response. KSHV evades immune cell detection by targeting antigen presentation mechanisms. To evade antigen recognition, KSHV encodes proteins such as K3 and K5 that ubiquitinate and degrade MHC‐I and MHC‐II molecules, while the vOX2 protein downregulates MHC‐II expression, all of which compromise both CD8⁺ and CD4⁺ T cell responses [230, 231, 232, 233]. Tumor biopsies from KS patients reveal spatial immune exclusion: CD8⁺ T cells are often localized away from KSHV‐infected regions despite high levels of chemoattractants [234]. Furthermore, CD4⁺ T cells and NK cells are largely absent from these areas, highlighting KSHV's potent ability to shape an immune‐privileged tumor microenvironment.

## 6 Models for Gammaherpesvirus Research

### 6.1 Challenges in Studying Human GHVs

Despite the clinical importance of EBV and KSHV, there are unique challenges for experimental research of these viruses. A central obstacle is the absence of robust model systems that can fully recapitulate human infection. These viruses exhibit a strict species tropism, in that they naturally infect only humans and human cells, making it difficult to study them in conventional animal models. Moreover, EBV and KSHV do not replicate efficiently in vitro, often persisting in cell culture as latent episomes rather than undergoing productive lytic replication. This limits the ability to study their complete life cycles. Adding to the challenge is the high global seroprevalence of EBV, which makes it difficult to obtain uninfected primary human samples for experimental infection studies.

Given these challenges, the development of physiologically relevant in vivo models has become a priority to bridge the gap between in vitro findings and human diseases. Recent advances have led to the creation of transgenic and humanized animal models tailored for GHV research [235, 236]. However, no single system fully mimics all aspects of GHV infection and pathogenesis in humans. A range of model systems, including immortalized cell lines, transgenic mice, humanized mice with tumor xenografts, and animal GHVs, have been developed, each offering distinct advantages and limitations depending on the specific research focus.

### 6.2 Cell Lines and Tissue Culture Models

Numerous established and engineered cell lines have significantly advanced our understanding of human GHV biology. These models facilitate dissection of viral molecular mechanisms under controlled conditions.

For EBV research, several B cell lines are widely used. Raji cells derived from BL, carry latent EBV that lack viral BALF2 and BARF1 genes essential for lytic replication, is exploited as a cell line for titrating fluorescently labeled infectious virus in what is known as the Green Raji Unit Assay [237, 238, 239]. Burkitt lymphoma cell lines can evict EBV in serial culture. BJAB cells, also derived from BL, are EBV‐negative, which serve as a host for infection of EBV recombinants and as isogenic negative controls [240]. EBV‐positive Akata and Mutu cell lines, both derived from BL, support virus reactivation, and are used as producer cells or to query mechanisms of reactivation [241, 242].

In addition, epithelial cell lines such as AGS (GC) and NPC lines are used to elucidate EBV's role in epithelial cell transformation and tumorigenesis [127, 243]. EBV immortalizes and transforms B‐cells and this property has provided the means to model PTLD, but attempts to immortalize epithelial cells with EBV have not been successful [244]. Furthermore, hTERT‐immortalized epithelial cells can retain EBV latent infection and grow as EBV recombinant cell lines, but do not form tumors in small animal models [245]. Thus, the growth of explanted NPC and GC‐derived tumor cell lines have been instrumental to mechanistic studies of EBV latency in cancer cells. A concerted effort to create tumor‐derived epithelial cell lines that retain native EBV infection, that are authenticated to be devoid of HeLa contaminants, remains a priority for cancer studies [246, 247, 248, 249]. Some of these tumor‐derived cell lines retain reactivation potential that can be used in targeted eradication studies by a kick and kill strategy [80, 250]. The addition of Rho‐associated coiled‐coil containing kinase (ROCK) inhibitor (Y‐27632) that suppress the epithelial differentiation has facilitated the creation of NPC cell lines [248]. Some of the tumor explants have been successfully passaged as patient‐derived xenografts (PDXs) [248]. Small animal models of NPC or GC are currently limited to sub‐cutaneous injections, which do not faithfully recapitulate mucosal immunity or the tumor microenvironment at the anatomical site of cancer presentation. It may be worthwhile pursuing oral orthotopic models as demonstrated in an NPC metastasis study [251]. Conversely, primary tonsillar B cells isolated from human tonsils, provide a physiologically relevant model to study primary EBV infection and latency establishment [252].

Because of the low replication efficiency and the inability to form plaques in culture, the study of EBV and KSHV infection and replication as well as generation of recombinant viruses has been particularly challenging. The cloning of the entire EBV and KSHV genomes into bacterial artificial chromosome (BAC) and generation of marker viruses have made these a possibility [253, 254]. The first KSHV BAC (BAC36) was used to delineate the functions of viral genes as well as cellular genes and pathways essential for KSHV infection and cellular transformation [254]. It was also used as a virus titration system based on the expression of the inserted GFP cassette in the infected cells [44]. The next generation of KSHV BACs, BAC16 and the modified BAC16‐RGB virus (with three stage‐specific reporters for latent, IE and L gene expression) have further advanced the field [255, 256]. While both EBV and KSHV researcers have developed BAC, CRISPR/Cas9, and transposon recombineering methods, there remains the challenge that genetic studies are limited to select viral isolates that do not capture the genetic diversity of circulating strains. Advances in CRISPR/Cas9‐mediated knock‐in of selectable genes into the GHV genome has provided one avenue of capturing viral isolates as molecular clones [257]. Nevertheless, the high copy number of viral genomes per cell introduces complexity, necessitating careful validation of experimental findings.

PEL cell lines are invaluable due to their direct association with KSHV infection and their ability to model KSHV‐driven oncogenesis. These cells derived from aggressive B‐cell lymphomas found in body cavities naturally harbor KSHV, with some also co‐infected with EBV [40, 166, 258]. The major PEL lines, including BC‐1 (KSHV+/EBV+), BC‐3 (KSHV+), BCBL‐1 (KSHV+), and BCP‐1 (KSHV+), have been instrumental in dissecting viral oncogenesis and studying KSHV‐EBV interactions [27, 259, 260]. However, they do not fully recapitulate the endothelial context of KS tumors. SLK and iSLK cells, originally thought to be immortalized from a KS lesion but now known to be contaminated with cells from a renal carcinoma cell line, are frequently used to model KSHV replication as they are highly permissive to KSHV replication upon induction [261]. iSLK cells can harbor latent KSHV‐BACs and support inducible lytic reactivation [262].

Primary HUVECs and telomerase‐immortalized human microvascular endothelial cells (TIME cells) are employed to study KSHV infection [44, 263]. Telomerase‐immortalized HUVECs (TIVE cells) and their KSHV‐infected cell clones KSHV‐TIVE are used to study KSHV‐induced oncogenesis [264]. However, given the observed low frequency of immortalization and transformation, and the possibility of harboring genetic alterations in the transformed cells, the usage of this system has been limited [264].

The presence of mesenchymal markers in KS tumor cells has long prompted investigators to consider mesenchymal precursor cells as the origin of KS tumor cells and good experimental models for KSHV infection and transformation [58, 265, 266]. Owing to the difficulty of transforming human primary cells, primary rat metanephric mesenchymal precursor cells (MM) were shown to be highly infectable by KSHV, which lead to rapid efficient cellular transformation [267]. These KSHV‐transformed cells (KMM) are primarily in a tight latent state and have been extensively used to delineate the functions of KSHV genes and cellular genes essential for cellular transformation [267]. These transformed cells can effectively induce KS‐like tumors in nude mice and express mixed cellular makers including vascular endothelial, lymphatic endothelial, and mesenchymal precursor markers that are hallmarks of KS tumors.

Following the work on the rat precursor cell model, human primary mesenchymal stem cells (hMSCs) were subsequently shown to be highly permissive to KSHV infection [58]. While the efficiencies of foci formation in cell culture and colony formation in semi‐soft agar are low, these infected cells also express a mix of KS markers and manifest angiogenic, invasive, and transforming phenotypes [58, 265, 266]. However, these cells fail to efficiently induce tumors in immunocompromised mice. KSHV can reprogram hMSCs to exhibit gene expression profiles similar to KS tumors under pro‐angiogenic conditions, emphasizing the importance of pro‐angiogenic signals in KSHV‐induced mesenchymal‐to‐endothelial transition of hMSCs [265]. More recent works show that KSHV‐infected endothelial colony forming cells (ECFCs) can induce KS‐like lesions with spindle cell morphology and high viral loads in immunocompromised mice [268]. However, the tumor induction efficiency of this model, which is critical for functional study, is unclear.

Beyond traditional monolayer cultures, advanced 3D tissue culture systems offer enhanced physiological relevance. These models allow for the investigation of complex cell‐cell and cell‐extracellular matrix (ECM) interactions critical for GHV pathogenesis [269]. For example, air‐liquid interface (ALI) cultures of epithelial cells simulate the mucosal architecture of the nasopharynx and oropharynx which are the natural sites of EBV entry and replication. In the upper airway, both pseudostratified airway epithelium and stratified epithelium can be modeled from conditionally reprogrammed cells of the nasopharynx, which enable the interrogation of de novo EBV infection [270, 271]. These models support epithelial differentiation and polarization, providing a valuable system to study viral entry, immune evasion, and epithelial cell tropism [272, 273]. ALI cultures were also used to examine KSHV lytic replication and gene expression [274]. Another breakthrough involves the use of organoids, which are 3D structures derived from stem or precursor cells that mimic the cellular diversity and function of specific tissues. Organoids enable long‐term culture and maintenance of multiple cell types, including epithelial, stromal, and immune cells, making them a versatile tool for modeling GHV‐host interactions in a tissue‐specific context [275].

For the purposes of this discussion, we have only highlighted key cell lines and tissue culture models that form the foundation of in vitro GHV research. In the following section, we will shift our focus to animal models and in vivo systems that complement and potentially extend these findings into translational applications.

### 6.3 Animal Models

There are three main types of animal models for human GHV research: experimental infection of laboratory animals, transgenic mice with humanized immune systems, and homologous animal GHVs studied in their natural hosts.

#### 6.3.1 Experimental Infection of Laboratory Animals

Direct experimental infection of animals with human GHVs offers the most straightforward route to study viral pathogenesis. However, it is also the most challenging due to species‐specific barriers to infection and disease manifestation. Among non‐human primates (NHPs), New World species such as cotton‐top tamarins and common marmosets are susceptible to EBV infection and can develop EBV‐associated lymphomas similar to those observed in humans [276]. KSHV infection of common marmosets has led to persistent infection in PBMCs, spleen, lymph nodes, and endothelium [277]. In rare cases, KSHV infection in marmosets has resulted in KS‐like lesions, though the low frequency and inconsistent development of these lesions limit its utility as a reliable KS tumorigenesis model [277]. Despite their limitations, such models are invaluable for examining disease progression and immune responses in a controlled setting. However, ethical considerations, high costs, and limited translatability due to interspecies differences constrain broader adoption of NHP models in GHV research.

#### 6.3.2 Humanized Mouse Models

Humanized mice, created by transplanting human hematopoietic stem cells (HSCs) into immunodeficient strains, such as NOD/Shi‐scid Il2rg^−/−^ (NOG, where NOD stands for non‐obese diabetes), Balb/c Rag2^−/−^ Il2rg^−/−^ (BRG), and NOD/LtSz‐scid Il2rg^−/−^ (NSG), allowed for the development of human immune components, including T, B, and NK cells, monocytes/macrophages, and DCs, provide a powerful system to study persistent GHV infections in a human‐like immune context [235, 236, 278, 279, 280, 281, 282, 283]. Human GHVs can infect and establish persistent infections in humanized mice, enabling the study of chronic infection, immune response, and viral pathogenesis in ways that are not possible in traditional laboratory animals. While they remain limited by the chimeric and transient nature of the reconstituted immune system, these models continue to evolve in sophistication.

Several other types of humanized models have been developed. The scid‐huPBL model involves injection of human peripheral blood leukocytes into SCID mice, whereas the scid‐huThy/Liv model uses fetal human thymus, liver, and lymph node tissue [278, 284, 285, 286]. The earlier models were constrained by short‐lived immune responses and susceptibility to graft‐versus‐host disease. Recent advances involve the transplantation of CD34^+^ hHSCs into NOD/SCID or NSG mice, enabling long‐term, multilineage reconstitution of the human immune system, including B, T, NK, and DCs, monocytes, and macrophages [287]. Similarly, scid‐huThy/Liv mice were transplanted with CD34^+^ hHSCs, combining the features of scid‐huThy/Liv mice and NOD/SCID mice [288]. These models have allowed EBV infection, replication, immune activation, and even lymphomagenesis with reconstituted MHC Classes I and II expression supporting more complete immune responses. BRG mice engineered with human cytokine genes (M‐CSF, IL‐3, GM‐CSF, thrombopoietin) exhibit enhanced myeloid and macrophage lineage development, supporting more robust innate immunity [289, 290, 291]. Intravenous infection of humanized mice with EBV triggers a rapid expansion of CD8^+^ T cells specific for viral antigens, mimicking the cellular immune response observed in IM. These mice often exhibit hepatosplenomegaly and elevated human cytokines, thereby more closely mimicking acute EBV infection. However, certain hallmarks of human infection, such as oral transmission are not recapitulated, likely due to the absence of compatible oropharyngeal epithelium expressing necessary viral entry receptors. Additionally, humanized mice do not exhibit GC‐dependent B cell differentiation due to an abnormal lymphoid structural microenvironment. For example, although human B and T cells are capable of contributing to GC reactions in the Bone marrow‐ Liver‐ Thymus‐ Spleen (BLTS) humanized mice, the absence of proper stromal architecture, particularly human follicular dendritic cells (FDC) networks, prevents the formation of functional GCs. As a result, GC‐dependent processes like affinity maturation, class switching, memory formation, and robust antibody production are significantly compromised [292]. These limitations prevent humanized mice from serving as fully representative models of infection.

A novel approach by Sin et al. [293] involved the generation of a transgenic mouse model for KSHV through pronuclear injection of the entire 170 kb KSHV genome. These immunocompetent mice developed aggressive angiosarcomas that histologically mimic KS. While KSHV latent genes were detected in non‐tumor endothelial cells, both latent and lytic genes were expressed in tumor tissues. Moreover, the tumors exhibited activation of hallmark KS‐associated pathways including PI3K/Akt/mTOR, IL‐10, and VEGF. However, this model does not reflect authentic KSHV infection dynamics as the virus is stably integrated into the mouse genome, thereby precluding studies of natural infection, replication, and horizontal transmission.

#### 6.3.3 Natural Animal GHV Homologs in Their Hosts

The third and arguably most biologically relevant approach leverages homologous GHVs in their natural animal hosts (Table 1). Old World NHPs such as rhesus macaques, harbor GHVs that are genetically and functionally analogous to EBV and KSHV [236, 277]. These NHPs have viruses that are inherently adapted to their host species but have genomic organizations and biological behaviors that mirror human GHVs, making them valuable models.

**Table 1:** Comparison of commonly used research models of animal‐GHV homologs for human GHV research.

Model virus	Rhesus lymphocryptovirus (RhLCV)	Rhesus rhadinovirus (RRV)	Retroperitoneal fibromatosis associated herpesvirus (RFHV)	Murine gammaherpesvirus 68 (MHV68)
Natural host	Rhesus macaques (*Macaca mulatta*)	Rhesus macaques (*Macaca mulatta*)	Macaques (various species)	Murid rodents, including lab mice (*Mus musculus*)
Related human virus	EBV	KSHV	KSHV	EBV and KSHV
Disease modeled	EBV‐associated lymphoproliferative disorders	Gammaherpesvirus pathogenesis and KS‐like lesions	Kaposi sarcoma‐like retroperitoneal fibromatosis	Latency, immune response, and lymphoproliferation
Genome homology with KSHV/EBV	High homology with EBV	High homology with KSHV, shares many oncogenic and immune‐modulatory genes	High homology with KSHV‐associated genes	Moderate homology
Infection outcomes	Persistent infection, lymphoproliferation	Persistent infection with KS‐like vascular lesions	Retroperitoneal fibromatosis (KS‐like tumors)	Latent infection, lymphoproliferative diseases, and splenomegaly
Latency	Primarily in B cells	Predominantly latent in B cells	Latent in B cells and endothelial cells	Primarily latent in B cells, also latent in macrophages and dendritic cells
Oncogenic potential	EBV‐like lymphomagenesis	Promotes tumorigenesis via vGPCR, vIL‐6, and other KSHV‐like oncogenes	Induces vascular tumor formation similar to KS	Causes lymphoproliferation, less associated with direct oncogenesis
Immune evasion	Encodes EBV‐like immune‐modulatory proteins	Encodes viral proteins homologous to KSHV immune evasion genes (e.g., vIL‐6, K3/K5)	Similar immune evasion mechanisms as KSHV	Modulates host cytokine responses, e.g., IFN suppression
Use in co‐infection models	Used with SIV to study EBV‐HIV interactions	Commonly used in SIV co‐infection studies to mimic KSHV‐HIV synergy	Limited co‐infection studies	Widely used in murine co‐infection models with other pathogens
Advantages	– Closely mimics EBV biology – Non‐human primate model	– Mimics KSHV biology – Non‐human primate model – Strong conservation of immune‐modulatory genes	– Closely models KS tumor biology – Models tumor angiogenesis	– Genetically tractable – Cost‐effective – Allows for studies in genetically modified hosts
Limitations	– Requires specialized facilities – Ethical concerns	– Differences from KSHV – Ethical and cost challenges	– Limited natural prevalence – Less characterized compared to RRV and MHV68	– No vascular lesions – Differences from human viruses
Applications	– Pathogenesis of EBV‐associated cancers – Vaccine and antiviral testing	– KSHV latency, immune evasion, and oncogenesis studies – Vaccine development for KSHV	– KS‐like tumor biology and angiogenesis studies – Testing of immunotherapies	– Latency, reactivation, and host‐virus interaction research – Preclinical antiviral screening

Rhesus lymphocryptovirus (rhLCV or Macacine gammaherpesvirus 4) is the closest analog to EBV. Genome‐wide analyses reveal high conservation of lytic‐cycle genes (49%–98% amino acid identity) and moderate conservation of latency‐associated genes (28%–60%) compared to EBV [294]. Like EBV, rhLCV establishes lifelong latent infection in memory B cells, transforms B cells in vitro, and is shed in saliva. Most captive rhesus macaques in standard housing become rhLCV‐seropositive by their first year, likely through oral transmission, highlighting the importance of rhLCV‐free colonies for in vivo research. Primary rhLCV infection via oral inoculation in seronegative rhesus macaques results in syndromes similar to acute EBV infection in humans, including atypical lymphocytosis, lymphadenopathy, and splenomegaly, followed by chronic asymptomatic infection [295]. This model recapitulates key immunovirological aspects of EBV pathogenesis and is ideal for dissecting both acute and latent infection mechanisms, and for testing vaccine candidates. However, whether the observed lymphocytosis in rhesus macaques results from virus‐specific CTL responses like IM remains to be clarified.

Rhesus rhadinovirus (RRV) and retroperitoneal fibromatosis‐associated herpesvirus (RFHV) are two homologs of KSHV [296, 297, 298]. These viruses provide valuable models to study KSHV‐like biology, particularly regarding latency establishment, lytic reactivation, immune evasion, and tumorigenesis. RRV infection in macaques mimics many aspects of KSHV biology and can be used to study GHV‐induced B cell lymphoproliferation and sarcoma‐like disease in immunocompromised settings such as co‐infection with simian immunodeficiency virus (SIV) [299, 300]. While RRV mimics many aspects of KSHV infection, differences in gene content and host‐specific responses limit its direct translational applicability. RFHV shares extensive genomic homology with KSHV and has been confirmed as the causative agent for RF, a KS‐like tumor, in macaques that presents with spindle cell features and inflammatory infiltration [297, 301, 302]. Experimental co‐infection of rhesus macaques with SIV and RFHV results in KS‐like lesions in the abdominal cavity rather than the skin [299]. However, the failure to isolate RFHV in culture and its incomplete characterization remain barriers to its use as a model system.

### 6.4 Murine Gammaherpesvirus 68 (MHV68) and Its Use as a Model Virus for GHV Studies

MHV68, also known as MuHV‐4, is the most widely used and experimentally tractable animal model for studying GHV infections [303]. A member of the *Rhadinovirus* genus, MHV68 naturally infects murid rodents and was first isolated from bank voles (*Clethrionomys glareolus*) in Slovakia in the early 1980s [304, 305]. It shares significant genetic and biological features with EBV and KSHV, and owing to its ability to infect inbred, outbred, and genetically modified mouse strains. Additionally, the MHV68 genome has been cloned into various BACs, allowing for easy mutagenesis of viral genes as well as use of various reporters [306, 307, 308]. The facile mutagenesis of the MHV68 genome, as well as the availability of multiple laboratory mouse genotypes, allows this system to serve as a powerful model to dissect the molecular mechanisms of GHV pathogenesis and host‐virus interactions [309].

**Figure 3:**
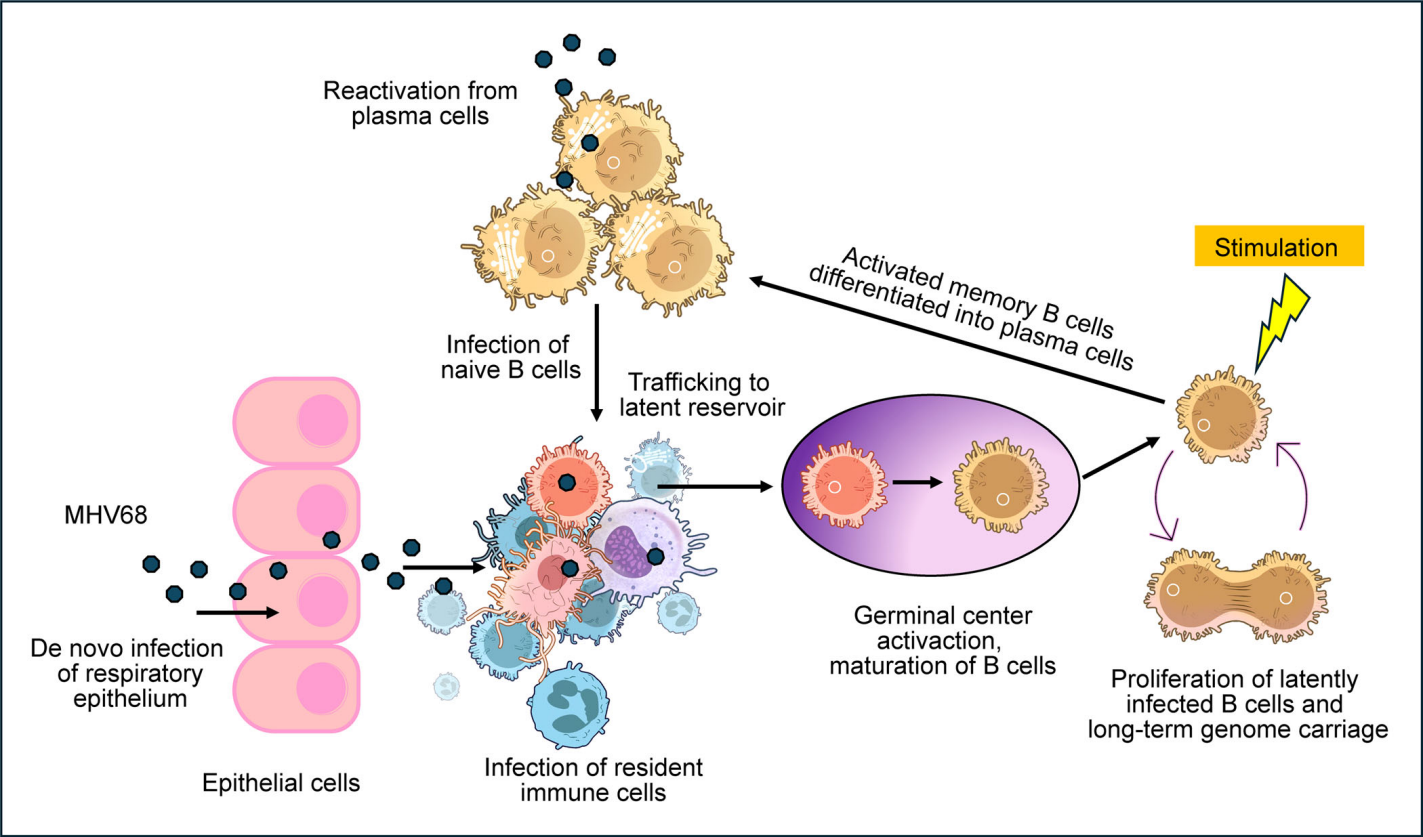
An illustrative model of the life cycle of the murine gammaherpesvirus 68. In laboratory infections, the virus first infects the respiratory epithelium. The virus then passes through LysM^+^ and CD11c^+^ myeloid cells, following which, lung‐resident B cells are infected. Lung‐resident immune cells of both lymphoid and myeloid origin are necessary for trafficking the virus to its reservoirs in the draining lymph nodes and spleen where the virus establishes latency in long‐lived memory B cells. Periodic homeostatic reactivation and polyclonal expansion of latently infected B cells maintains the latency load through the life of the host, while differentiation of infected B‐cells into plasma cells releases progeny virions. This figure was created on Adobe Illustrator with icons sourced from the National Institute of Allergy and Infectious Diseases, National Institute of Health Bioart Source.

MHV68 exhibits a typical biphasic herpesvirus life cycle, alternating between lytic replication and latency [310]. In the lytic phase, the virus undergoes rapid replication and dissemination, whereas during latency it persists in host cells, primarily B lymphocytes, remaining largely silent and shielded from immune detection (Figure 3). This latent state allows lifelong persistence, a hallmark of herpesvirus biology. MHV68 primarily infects B cells, macrophages, and epithelial cells. Upon de novo infection in epithelial cells, it enters the lytic phase marked by a temporally regulated gene expression cascade [310] with RTA initiating this cascade [311]. Additional IE genes include ORF73 (LANA), a homolog of KSHV's LANA, and ORF57, which encodes the mRNA transport activator (MTA) [310, 311, 312]. These genes precede early gene expression for viral DNA replication and late gene expression for structural proteins. Following virion assembly, the virus exits the host cell by lysis.

Although there have been rare reports of MHV68 infection of human cell lines in vitro, there is no evidence of MHV68 infection in humans to date [313], suggesting that MHV68 poses a substantially lower occupational risk compared to human GHVs. MHV68 effectively models many aspects of GHV biology, including immune evasion, latency, and reactivation. In natural hosts, MHV68 infection typically causes mild to moderate, non‐fatal symptoms and establishes latency independent of route and dose of infection [314]. In laboratory mice, MHV68 infection can lead to pathologies reminiscent of human GHV‐related diseases, including LPDs and symptoms similar to IM, such as CD8⁺ T cell lymphocytosis, polyclonal B cell activation with autoantibody production, and splenomegaly [315]. Moreover, the IM‐like disease seen in adult mice post‐infection is not observed in neonates, mirroring EBV's age‐dependent symptomatology [316].

MHV68's tropism for B cells provides an invaluable model for probing B cell involvement in viral persistence and pathogenesis. It encodes homologs of KSHV genes implicated in pathogenesis and latency, including LANA, v‐cyclin (ORF72), v‐Bcl‐2 (M11), vGPCR (ORF74), and the complement control protein (ORF4), and like KSHV, MHV68 induces B cell lymphoproliferative disease in immunocompromised mice [317]. In addition to lymphoproliferation, MHV68 infection has been associated with respiratory, hepatic, and other morbidities under experimental conditions [184, 318]. These features underscore its utility for studying GHV latency, immune control, and reactivation, though its limitations in modeling GHV‐associated malignancies must also be acknowledged, as discussed below.

Unlike EBV, which exhibits multiple latency programs, MHV68 and KSHV exhibit a single latency program characterized by limited gene expression and episomal persistence of the viral genome, predominantly in B cells as well as precursor cells for KSHV (Figures 2 and 3). Similar to KSHV, MHV68 LANA ensures the maintenance and partitioning of episomes during B cell division [308, 319]. The latency‐associated M2 gene promotes germinal center reactions in the spleen and lymph nodes, facilitating B cell differentiation into plasma cells or memory B cells with the latter serving as long‐term viral reservoirs [317, 320]. Interestingly, aberrant germinal center B cell expansion is restricted by activation of the tumor suppressor p53 [321]. While the long‐lived memory B cells provide a stable host for the latent virus, differentiation into plasma cells triggers virus reactivation and restarts the entire biphasic life cycle [322, 323]. At this point, the virus expands its reservoir by infecting adjacent naïve B cells or facilitates transmission to new hosts. Expansion of the latent viral reservoir may also occur via polyclonal proliferation of infected B cells without reactivation [307].

Examination of the immune response to MHV68 has provided us with many clues regarding the immune response to human GHV infections. MHV68 infection triggers robust immune responses. GHV lytic replication induces tissue damage and elicits innate immune responses involving interferons and cytokines such as IL‐6, IL‐10, and IFN‐γ [184, 194]. Type I IFNs are essential for controlling acute infection, latency establishment, and reactivation. IRF3 is a key driver of Type I IFN expression in macrophages during lytic infection through IFNAR‐dependent signaling [324, 325]. In Ifnar1^−/−^ mice deficient in Type I IFN signaling, MHV68 shows increased propagation, earlier systemic dissemination, and heightened reactivation from latently infected splenocytes [326]. Type I IFN‐deficient mice survive only low‐dose MHV68 infections [325]. While wild type mice effectively manage acute infection, Ifnar1^−/−^ mice display dose‐dependent susceptibility and faster systemic dissemination [326]. Ifnar1^−/−^ mice also display impaired cytokine responses (TNF‐α, IFN‐γ, IL‐2) in CD8⁺ T cells [326].

Type I IFN signaling indirectly regulates CD8⁺ T cells by controlling viral replication and T cell‐specific disruption of this pathway does not hinder their function [326]. Additional inflammatory pathways promote T cell trafficking to infected tissues. IRF1, which is upregulated by IFN‐β, plays a critical role in limiting germinal center activity and controlling the latent reservoir by modulating T cell subsets. IRF1 deficiency enhances IL‐17A–producing CD4⁺ T cells and follicular helper T cells, expanding the latent pool and germinal center reactions in infected mice [327].

TLR signaling plays a critical role in the immune response to MHV68 infection [194, 328]. Among these, TLR9 is a key sensor of MHV68, triggering the production of IFN‐α, IL‐6, and IL‐12 by DCs, and contributing to reduced viral burden in the spleen following intraperitoneal infection. TLR9 also mitigates MHV68‐induced lung fibrosis and promotes Type I IFN production in the lungs during intranasal infection [194]. Combined deletion of TLR7 and TLR9 in plasmacytoid DCs abolishes IFN‐α secretion, highlighting their cooperative roles in sensing MHV68 and initiating antiviral immunity [329].

CD4^+^ T cells are indispensable for controlling persistent GHV infection, and it also holds true for MHV68. A heterogeneous population of CD4^+^ T helper cell clones arises during infection, likely activated by uninfected myeloid APCs that present MHV68‐derived peptides on MHC Class II molecules [184]. CD4^+^ and CD8^+^ T cells act synergistically to limit viral replication. For instance, in the absence of CD4^+^ T cells, MHV68‐infected mice develop a subset of CD8^+^ T cells that produce IL‐10, thus suppressing anti‐viral immune surveillance [330]. Further studies are needed to fully elucidate how CD4^+^ and CD8^+^ T cell responses are regulated to achieve durable viral control while minimizing immunopathology [184].

MHV68's immune evasion tactics include downregulating MHC molecules and disrupting cytokine signaling. KSHV, on the other hand, employs a diverse array of immune modulators, such as vFLIP, which inhibits apoptosis [331], and vIRFs, which suppress Type I interferon signaling [228]. The Type I IFN response is a cornerstone of host defense against GHVs, including MHV68, and is crucial for both acute and latent phase control. To evade this response, MHV68 has evolved multiple strategies to interfere with IFN signaling pathways. For instance, the viral protein ORF64 mediates the efficient delivery of viral DNA to the nucleus, thereby evading cytosolic DNA sensing mechanisms [332, 333]. Additionally, ORF11 inhibits IRF3‐driven Ifnb1 transcription by disrupting TBK1‐IRF3 interactions [334], and ORF36 blocks IFN‐β production by preventing recruitment of RNA polymerase II to the Ifnb1 promoter through nuclear interference with activated IRF3 [325].

Though genetically related, MHV68 and KSHV differ in their genomic content. MHV68 harbors unique M genes that encode immunomodulatory proteins, while KSHV encodes several human gene homologs, such as vIL‐6, that contribute to inflammation, angiogenesis, and immune evasion. The M2 latency‐associated protein further suppresses IFN‐I signaling by downregulating STAT1 and STAT2 in both fibroblasts and B lymphocytes [184, 325].

MHV68, like KSHV, also employs various immune evasion strategies that impair CD8^+^ T cell‐mediated antiviral responses. Among the most impactful are viral proteins that suppress antigen presentation or immune effector function. The MHV68 and KSHV genome maintenance proteins (GMPs) contribute to both the persistence of latency and evasion of CD8^+^ T cell immunity [335]. The KSHV K3 and K5 proteins downregulate MHC Class I glycoproteins and while the MHV68 M3 protein neutralizes host chemokines [230, 336]. Furthermore, infection of the thymus by GHV may alter thymocyte development, potentially leading to central tolerance of viral epitopes and preemptive deletion of virus‐specific CD8^+^ T cells before peripheral activation [184].

While murine models of MHV68 infection have yielded invaluable insights into GHV biology by recapitulating various phenomena seen in KSHV or EBV infections, several limitations should be acknowledged. For instance, MHV68 latency expansion in GC B cells requires CD4^+^ T_fh_ help, in contrast to EBV which is capable of bypassing CD4^+^ T_fh_ cell driven B cell proliferation by expressing viral homologs of T_fh_ signals [337]. MHV68 genes do not always have direct homologs in human GHVs, and immune responses differ between species. Moreover, MHV68 infects a broader range of cell types than EBV or KSHV, limiting its utility for modeling tissue‐specific pathogenesis of human viruses. Despite these limitations, advances in reverse genetics and recombinant virus technologies have enabled detailed functional delineation of viral genes in in vivo infection models that are not possible with human GHVs. BACs have facilitated the generation of MHV68 mutants with targeted deletions, insertions, or substitutions, and have been instrumental in expressing KSHV homologs in MHV68 for comparative analysis [338, 339]. These systems have been pivotal in delineating the roles of specific genes in viral replication, latency establishment, and maintenance.

## 7 Perspectives and Conclusion

GHVs play critical roles in tumor virology, with their ability to drive oncogenesis through persistent infection and immune evasion offering deep insights into cancer biology and viral pathogenesis. Comprehensive studies of host‐pathogen interactions, especially the immune responses that shape GHV pathogenesis, have been instrumental in our understanding of various diseases.

Multiple model systems have played important roles in dissecting the biology of GHV infection and disease progression; and over the past few decades we have seen much progress in developing newer and better models for examining distinct aspects of GHV biology. Each model has its own strengths and limitations, and when selecting a suitable model, researchers must consider multiple factors including feasibility and cost. While NHPs offer the closest genetic and physiological parallels to humans and their GHVs serve as highly relevant models, such studies are often limited to institutions with significant financial resources. Additionally, public opinion of primate research is seldom favorable, and justifying the work of primate research centers to a lay audience is often an arduous task. In contrast, murine models, particularly *Mus musculus*, provide a more accessible (and easier to justify) alternative. Among these, MHV68 remains a valuable and widely used model for studying GHV infection.

MHV68 can establish both latent and lytic infections in mice, recapitulating many key features of human GHV infection, including immunological interactions and host responses. It provides a valuable platform for the investigation of latency establishment, reactivation, and viral persistence under physiological conditions, challenges that are difficult to overcome in human or in vitro systems.

However, MHV68 does not fully replicate the biology of human GHVs. Differences in tropism, gene expression, and latency programs remain notable. For instance, MHV68 primarily targets B cells in the spleen and lymph nodes, whereas KSHV persists in circulating B cells and precursor cells. The differences in immune responses between mice and humans also present challenges, as certain immune evasion strategies employed by human viruses may not be fully mirrored in MHV68 infections. Nevertheless, MHV68 offers critical insights into immune evasion strategies shared with human GHVs. It interacts with a range of different immune cells, including B cells and macrophages, and other immune components, providing a platform to study immune modulation and viral persistence.

Despite these strengths, several important avenues of GHV research remain underexplored. For instance, the influence of nutrient availability, metabolic states, and lipid signaling on GHV latency and reactivation is poorly understood although the roles of metabolic pathways in human GHV replication and latency have been defined in cell culture models [340, 341]. Likewise, the extent to which murine and human GHV‐induced pathologies overlap remains an open question. Most studies rely on genetically homogeneous mouse strains, limiting our ability to uncover host‐specific factors that influence infection outcomes. The use of genetically diverse mouse populations such as Collaborative Cross Mice could provide new insight into host genetic contributions to susceptibility, latency control, and disease progression as they have in the case of other viruses [342].

While current genetic tools allow examination of individual viral genes, broader systems‐level approaches are needed to capture the complexity of host‐virus interactions. Recent advances in systems biology and multi‐omics approaches like spatial in‐situ, single‐cell transcriptomics, and proteomics can offer unprecedented resolution for investigating GHV‐host interactions. These tools can uncover cell‐type‐specific viral programs and immune responses, helping to clarify the complex orchestration of latency and reactivation. Additionally, MHV68 offers a platform to study co‐infections and microbiome interactions, an emerging area of interest as we begin to appreciate how microbial ecology and polymicrobial infections may influence GHV pathogenesis [343].

Finally, the knowledge gained from MHV68 models is extremely relevant to translational goals. Understanding how GHVs modulate the immune system may help in the design of vaccines, latency‐targeting antivirals, and immunotherapies. By identifying viral and host factors that control latency and reactivation, MHV68 studies can aid in the development of strategies to prevent GHV‐associated diseases in immunocompromised populations.

In summary, while MHV68 cannot fully replicate human GHV infection, it remains an indispensable model for uncovering conserved mechanisms of latency, immune evasion, and pathogenesis. Continued integration of emerging technologies into the study of animal models will be instrumental in bridging existing knowledge gaps and advancing both basic virology and translational research against these pathogens.

## Author Contributions

Arundhati Gupta analyzed and organized data, wrote original draft, reviewed and edited manuscript. Renfeng Li wrote original draft, reviewed and edited manuscript. Kathy Shair wrote original draft, reviewed and edited manuscript. Shou‐Jiang Gao conceptualized this study, secured funding, provided supervision, analyzed and organized data, wrote original draft, reviewed and edited manuscript.

## Conflicts of Interest

The authors declare no conflicts of interest.

## Data Availability

The data that support the findings of this study are available on request from the corresponding author. The data are not publicly available due to privacy or ethical restrictions.
